# Regulation of β-catenin by t-DARPP in upper gastrointestinal cancer cells

**DOI:** 10.1186/1476-4598-10-32

**Published:** 2011-03-29

**Authors:** Bhavatarini Vangamudi, Shoumin Zhu, Mohammed Soutto, Abbes Belkhiri, Wael El-Rifai

**Affiliations:** 1 Department of Surgery, Vanderbilt University Medical Center, Nashville, Tennessee, USA; 2 Department of Cancer Biology, Vanderbilt University Medical Center, Nashville, Tennessee, USA

## Abstract

**Background:**

Truncated dopamine and cyclic-AMP-regulated phosphoprotein (t-DARPP) is frequently overexpressed in gastrointestinal malignancies. In this study, we examined the role of t-DARPP in regulating β-catenin.

**Results:**

The pTopFlash construct that contains multiple TCF/LEF-binding sites was used as a measure of β-catenin/TCF transcription activity. Gastric (AGS, MKN28) and esophageal (FLO-1) adenocarcinoma cancer cell lines that lack t-DARPP expression were utilized to establish stable and transient *in vitro *expression models of t-DARPP. The expression of t-DARPP led to a significant induction of the pTOP reporter activity, indicative of activation of β-catenin/TCF nuclear signaling. Immunofluorescence assays supported this finding and showed accumulation and nuclear translocation of β-catenin in cells expressing t-DARPP. These cells had a significant increase in their proliferative capacity and demonstrated up-regulation of two transcription targets of β-catenin/TCF: Cyclin D1 and c-MYC. Because phosphorylated GSK-3β is inactive and loses its ability to phosphorylate β-catenin and target it towards degradation by the proteasome, we next examined the levels of phospho-GSK-3β. These results demonstrated an increase in phospho-GSK-3β and phospho-AKT. The knockdown of endogenous t-DARPP in MKN45 cancer cells demonstrated a reversal of the signaling events. To examine whether t-DARPP mediated GSK-3β phosphorylation in an AKT-dependent manner, we used a pharmacologic inhibitor of PI3K/AKT, LY294002, in cancer cells expressing t-DARPP. This treatment abolished the phosphorylation of AKT and GSK-3β leading to a reduction in β-catenin, Cyclin D1, and c-MYC protein levels.

**Conclusions:**

Our findings demonstrate, for the first time, that t-DARPP regulates β-catenin/TCF activity, thereby implicating a novel oncogenic signaling in upper gastrointestinal cancers.

## Background

Upper gastrointestinal adenocarcinomas (UGCs) are among the most prevalent causes of cancer-related deaths in the world. This category of cancers includes adenocarcinomas of the stomach, gastroesophageal junction (GEJ), and lower esophagus. While gastric carcinomas remain the world's second leading cause of cancer-related deaths [1,2], the incidence and prevalence of adenocarcinomas of the esophagus and GEJ has dramatically increased amongst the Western population [3-6]. The biology of gastrointestinal cancer involves complex signaling mechanisms and critical molecular interactions, most of which remain uncharacterized [7-9]. Although chemotherapy is currently one of the primary options for treatment of gastric cancer, it often provides poor clinical prognosis due to the underlying resistance mechanisms [10,11]. Limited understanding of such inherent protective mechanisms enforces a need to identify novel signaling pathways that can possibly reveal novel drug targets towards the development of advanced therapeutic alternatives. Dopamine and cyclic-AMP-regulated phosphoprotein (DARPP-32), also known as PPR1R1B, is a major regulator of dopaminergic neurotransmission in the brain and is the key factor for the functioning of dopaminoceptive neurons [12]. Molecular investigation of critical target genes at 17q12 amplicon in gastric adenocarcinoma has led to the identification of DARPP-32 and t-DARPP, a truncated isoform of DARPP-32, as two novel cancer-related genes [13]. t-DARPP is frequently overexpressed in several human adenocarcinomas such as those of the stomach, colon, esophagus, breast, and prostate [14-18]. However, the molecular signaling mechanisms governing t-DARPP's biological functions remain fairly unexplored.

Wnt signaling is one of the most critical pathways for regulation of cell proliferation, differentiation and migration during embryonic patterning and morphogenesis [19-21]. One of the key events of canonical or Wnt/β-catenin-dependent pathways is accumulation and nuclear translocation of β-catenin, which is an integral component of adherens junctions [22-24]. Dysregulation and aberrant activation of Wnt pathways or mutations in β-catenin or adenomatous polyposis coli (APC) often results in increased β-catenin accumulation. The oncogenic potential of nuclear β-catenin in the initiation and progression of various human malignancies including carcinomas of colon and esophagus have been discussed [25-29]. Glycogen synthase kinase-3β (GSK-3β) plays an important role in determining β-catenin turnover inside the cells. In the absence of Wnt/Wingless ligand activation, β-catenin exists in the cytoplasm as a multi-protein complex with scaffold protein Axin, APC, PP2A (protein phosphatase 2A), GSK-3β, and CK1 (casein kinase I) [30-35]. When this destruction complex is intact, GSK-3β phosphorylates the amino terminal serine and threonine residues of β-catenin and targets it towards degradation by proteasomal machinery [36-38]. The phosphatidylinositol 3-kinase (PI3K)/AKT signaling pathway is a major regulator of GSK-3β [39,40]. AKT-mediated phosphorylation and inactivation of GSK-3β leads to hypophosphorylation and stabilization of cytosolic β-catenin with subsequent accumulation and translocation into the nucleus. In the nucleus, β-catenin functions as a transcriptional co-activator of the T-cell factor/lymphoid enhancer factor (TCF/LEF) family of DNA-binding transcription factors [41-43]. This complex binds to and activates several Wnt target genes including c-MYC, Cyclin D1, MDR1, and VEGF many of which are involved in tumorigenesis [44-47]. In this study, we have reported that t-DARPP can regulate β-catenin/TCF signaling in upper gastrointestinal cancer cells.

## Results

### Activation of β-catenin/TCF reporter and nuclear localization of β-catenin by t-DARPP

**Figure 1:**
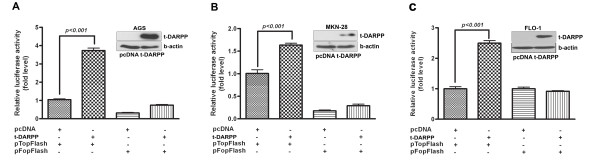
**t-DARPP regulates β-catenin/TCF activation**. β-catenin and TCF/LEF transcriptional activity was assessed using the luciferase reporter constructs, pTopFlash (with six TCF binding sites) and pFopFlash (with mutant binding site). AGS (A) MKN28 (B) and FLO-1 (C) cells that lack the expression of endogenous t-DARPP, were transiently transfected with different plasmid constructs as shown. The Western blot insets demonstrate the level of t-DARPP in representative transient transfection experiments. Overexpression of t-DARPP resulted in significant induction of β-catenin activity (p < 0.001) as indicated by an increase in luciferase activity of pTopFlash reporter. Co-transfection of t-DARPP pFopFlash reporter (mutant reporter) did not show any effect on luciferase activity. Results are representative of at least three experiments and shown as the mean with ± SD. Significance of difference was calculated using one-way *ANOVA*.

**Figure 2:**
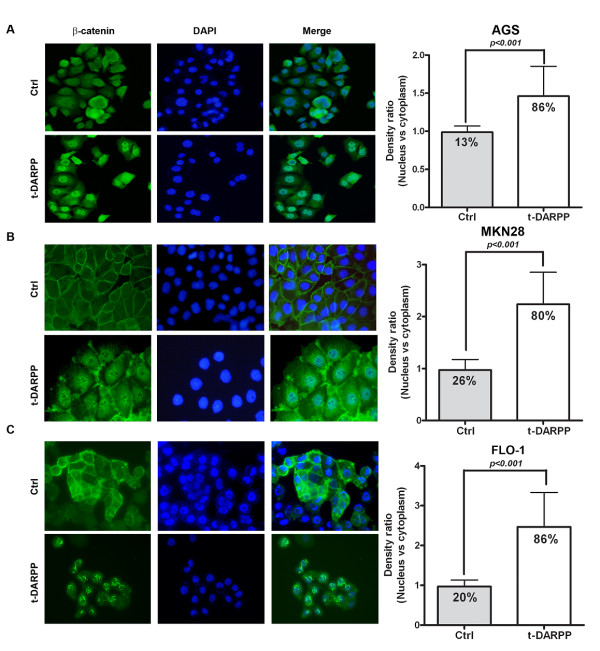
**t-DARPP increases nuclear accumulation of β-catenin**. Immunofluorescence analysis was performed on AGS (A) MKN28 (B) and FLO-1 (C) cells overexpressing t-DARPP. Data indicates increased nuclear localization of β-catenin in cells expressing exogenous t-DARPP, as compared to control cells that show membranous β-catenin (p < 0.001). β-catenin positive cells were analyzed by using an anti-β-catenin antibody which is recognized by secondary rabbit antibody conjugated with FITC, depicted by green fluorescence. Nuclear staining was detected by counterstaining cells with 4', 6-Diamidino-2-phenylindole (DAPI), represented as blue fluorescence. Ratio of cells positive for nuclear β-catenin staining to total number of cells was counted as percentage positive for nuclear localization. In total, at least 300 cells from t-DARPP and control vector were counted from three different microscopic fields for β-catenin immunofluorescence. Results are representative of three independent experiments and expressed as mean values ± SD. Significance of difference was calculated using Student's t test.

We utilized the β-catenin reporter assays using both the pTopFlash construct, which contains six functional TCF/LEF-binding sites in the promoter of a firefly luciferase reporter gene, and the derived pFopFlash construct with mutated TCF/LEF-binding sites. The transient transfection of t-DARPP in AGS, MKN28 and FLO-1 cells that lack endogenous t-DARPP led to 3.5, 1.5, and 2.5 fold induction (*p < 0.001*) in the pTopFlash reporter activity relative to control pcDNA3 in AGS, MKN28, and FLO-1, respectively (Figure 1). The specificity of β-catenin/TCF was confirmed by the co-transfection of different expression vectors with mutant pFopFlash reporter. In line with these findings, the immunofluorescence studies indicated a significant increase (*P < 0.001*) in the percentage of cells showing accumulation and nuclear localization of β-catenin in cells transfected with t-DARPP as compared to empty vector control; AGS (86% vs 13%) and MKN28 (80% vs 26%) gastric cancer cells and FLO-1 (86% vs 20%) (Figure 2). These results augment the findings of the reporter assays and strongly suggest the possible role of t-DARPP in mediating accumulation and nuclear translocation of β-catenin and activation of β-catenin/TCF transcription complex.

### t-DARPP increases the proliferative capacity of gastric cancer cells

**Figure 3:**
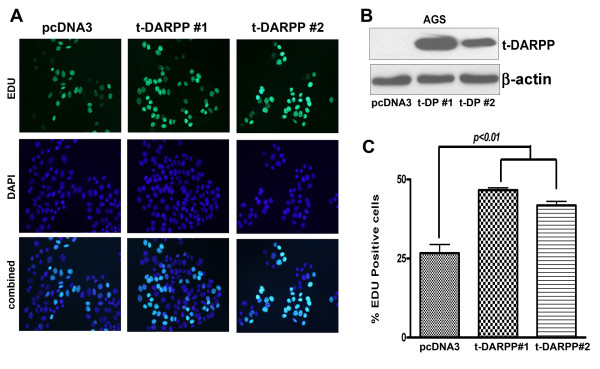
**Enhanced cell proliferation of gastric cancer cells overexpressing t-DARPP**. (A) AGS cells stably transfected with t-DARPP (clones #1 and #2) demonstrate an increased rate of cell proliferation (48% and 45%) as compared to pcDNA empty vector control cells (26%, p < 0.01). ClickiT^® ^EdU Assay (Invitrogen) was utilized to measure cell proliferation in t-DARPP expressing cells. EdU (5-ethynyl-2'-deoxyuridine) is incorporated as a thymidine analog during active DNA synthesis. EdU labeling in cells is detected by the binding of azide group of Alexa Fluor 488 dye (depicted as green fluorescence) to alkyne group of EdU. Ratio of EdU positive cells to total number of cells (represented by blue nuclei stain, DAPI) is a direct index of the number of proliferating cells. (B) t-DARPP overexpression in AGS stable clones is demonstrated by Western blot from total proteins. (C) Quantification data represents results obtained from two independent t-DARPP stable clones (#1 and #2). 500 cells were counted for each experiment and the percentage of EdU positive cells for each clone was averaged from at least four independent microscopic fields. Results are representative of at least three independent experiments and shown as mean ± SD. Significance of difference was calculated using one-way *ANOVA*.

One of the important functions of β-catenin signaling in cancer is the promotion of cellular proliferation. Using an EDU proliferation assay and counting 500 cells from each experiment, we showed that 45-48% of AGS cells stably transfected with t-DARPP (clones #1 and #2) demonstrate nuclear EDU staining (green fluorescence) whereas only 26% of control cells showed a similar staining (*p < 0.01*). These results were corroborated in two independent AGS clones stably expressing t-DARPP showing a significant increase in the number of cells with nuclear EDU staining, indicative of increased cell proliferation (Figure 3).

### t-DARPP expression up-regulates β-catenin and induces its targets

**Figure 4:**
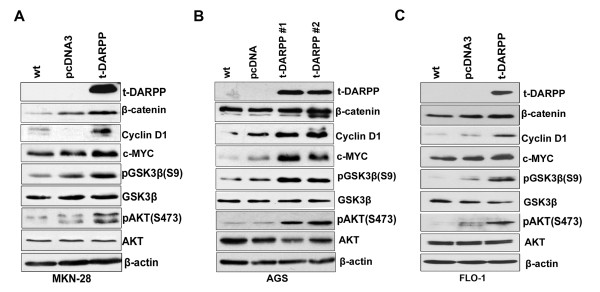
**t-DARPP overexpression leads to up-regulation of β-catenin and its downstream targets**. Proteins extracted from MKN28 cells stably expressing t-DARPP (pool) (A) and AGS t-DARPP stables (clones #1 and #2) (B) FLO-1 cells stably expressing t-DARPP (pool) (C) were subjected to immunoblot analysis using t-DARPP, β-catenin, c-MYC, Cyclin D1, GSK-3β, pGSK-3β (Ser9), AKT, pAKT (Ser473), and Actin antibodies. Protein loading was normalized to equal levels of β-actin. Total protein levels of β-catenin and its targets, c-MYC and Cyclin D1, were significantly higher in t-DARPP expressing cells compared to control cells. Both pGSK-3β (Ser9) and pAKT (Ser473) were higher in t-DARPP expressing cells as compared to control.

**Figure 5:**
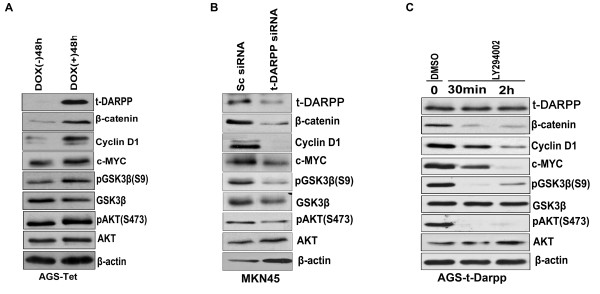
**Regulation of β-catenin by t-DARPP is AKT-dependent**. (A) t-DARPP expression was induced in tetracycline-inducible AGS-t-DARPP cells following treatment with doxycycline for a period of 48 h. Consistent with findings in cells stably overexpressing t-DARPP, induction of t-DARPP expression led to marked accumulation of β-catenin, c-MYC, Cyclin D1, pGSK-3β (Ser9), and pAKT (Ser473). (B) Proteins obtained from MKN45 cells that express endogenous t-DARPP transfected with either control scrambled siRNA or t-DARPP specific siRNA oligonucleotides were subjected to Western blot analysis. As shown, knockdown of endogenous t-DARPP led to a marked decrease in protein levels of β-catenin, c-MYC, Cyclin D1, pAKT (Ser473), and pGSK-3β (Ser9). (C) AGS cells stably overexpressing t-DARPP were treated with dimethyl sulfoxide (DMSO) as control and LY294002 (40 uM), a potent PI3 kinase inhibitor, for 30 min and 2 h. As shown by Western blot analysis, treatment with LY294002 led to complete abrogation of downstream AKT and GSK-3β phosphorylation in t-DARPP expressing AGS cells. Inhibition of PI3 kinase in AGS-t-DARPP cells resulted in significant downregulation of β-catenin, c-MYC and Cyclin D1.

Accordant with our immunofluorescence results, Western blot analysis in cells stably expressing t-DARPP showed an increase in the protein levels of β-catenin in AGS and MKN28 gastric cancer cells and FLO-1 esophageal cancer cells (Figure 4). Consistent with reports that identified c-MYC and Cyclin D1 as two important targets of the β-catenin/TCF transcription complex [19,33,44,48], our results demonstrated that t-DARPP-mediated activation of β-catenin/TCF leads to up-regulation of c-MYC and Cyclin D1 in gastric and esophageal cancer cells (Figure 4). These results explain the observed increase in the proliferative capacity in t-DARPP expressing cells (Figure 3). In line with the role of active GSK-3β in regulating β-catenin degradation [19,49], our results indicated an increase in the phosphorylation levels of GSK-3β (Ser 9), indicative of the loss of GSK-3β activity (Figure 4). The PI3K/AKT signaling is one of the most fundamental pathways for cell proliferation and is frequently linked to human cancer [50-52]. Western blot analysis indicated that phosphorylation of AKT at Ser473 was remarkably higher in AGS, MKN28 and FLO-1 cells stably expressing t-DARPP as compared to the control cells (Figure 4), thus providing an explanation for the increase in GSK-3β (Ser9) phosphorylation. Phosphorylated GSK-3β loses its ability to phosphorylate and target β-catenin towards degradation by proteasomes, resulting in accumulation and translocation of β-catenin to the nucleus [19,49]. Furthermore, we confirmed our observations by using tet-inducible AGS cells expressing t-DARPP. Induction of t-DARPP expression by treatment with doxycycline for 48 h led to a significant induction of β-catenin protein levels (Figure 5A). To ascertain the role of t-DARPP in the regulation of β-catenin levels via GSK-3β phosphorylation, we used t-DARPP specific siRNA to knockdown endogenous t-DARPP (MKN45 cells). The knockdown of t-DARPP led to a remarkable decrease in the levels of phosphorylated GSK-3β, β-catenin, c-MYC, and Cyclin D1 (Figure 5). In order to confirm t-DARPP-mediated regulation of TCF/β-catenin activity via PI3K/AKT pathway, we used pharmacologic inhibition of PI3K/AKT on AGS cells stably expressing t-DARPP. Our data demonstrate a significant abrogation of pAKT (Ser473) and pGSK-3β (Ser9) after treatment with LY492002 for 30 min and 2 h (Figure 5C). This treatment also dramatically reduced levels of β-catenin and its targets c-MYC and Cyclin D1 (Figure 5C). Taken together, our findings suggest the possible role of t-DARPP in regulating the cross-talk between PI3K/AKT and Wnt/β-catenin pathways in gastric carcinogenesis.

## Discussion

t-DARPP has been recently identified as a splice variant of DARPP-32 [53]. Both DARPP-32 and t-DARPP genes are located at the 17q12 locus, a region frequently amplified in gastrointestinal adenocarcinomas [13,54,55]. Although DARPP-32 has been known as a major regulator of dopamine signaling in the central nervous system [56,57], the functions of DARPP-32 and t-DARPP in cancer remain largely unexplored. Our previous results indicated that t-DARPP-induced cell proliferation is possibly mediated by c-MYC and Cyclin D1 [58]. These findings suggested the possible role of t-DARPP in regulating Wnt/β-catenin signaling in cancer cells. In this study, we have identified and confirmed a novel function of t-DARPP in regulating Wnt/β-catenin signaling in upper gastrointestinal cancer cells.

Wnt signal transduction pathway is by far one of the most important pathways for regulation of cell proliferation, differentiation, migration, and survival/apoptosis. Alterations in β-catenin signaling are a common finding in several cancers [59,60]. Using the β-catenin/TCF luciferase reporter (pTopFlash) to measure the activation of β-catenin/TCF complex, t-DARPP increased the activity of this reporter in gastric and esophageal cancer cell models. The activity of Wnt/β-catenin signaling pathway depends on the accumulation and translocation of β-catenin to the nucleus, one of the important factors for the initiation of tumorigenesis in a variety of human cancers [25-27]. Accumulation and nuclear localization of β-catenin have been reported in approximately one-third of gastric tumors [25,61,62]. Immunofluorescence analysis on t-DARPP expressing cells showed remarkable accumulation of nuclear β-catenin. In the nucleus, the β-catenin/TCF transcription complex regulates the expression of several genes that are involved in human carcinogenesis such as Cyclin D1 and c-MYC [25,60-65]. The *in vitro *cell models expressing t-DARPP demonstrated up-regulation of Cyclin D1 and c-MYC protein levels. This finding was associated with increased proliferation in t-DARPP expressing cells as compared to empty vector control. Taken together, our findings provide strong evidence that t-DARPP plays a role in nuclear translocation of β-catenin and oncogenic induction of β-catenin/TCF transcriptional activity, the outcome of which is reflected in the increased proliferation capacity. In an attempt to determine the underlying signaling mechanism by which t-DARPP regulates β-catenin, we demonstrated that t-DARPP overexpression in gastric and esophageal cancer cells was associated with increased phosphorylation of GSK-3β. GSK-3β plays a critical role in Wnt/β-catenin signaling by regulating the levels of cytoplasmic β-catenin. GSK-3β is rendered inactive by phosphorylation resulting in accumulation and nuclear translocation of non-phosphorylated β-catenin [20,21]. Consistent with these studies, we detected a remarkable up-regulation in β-catenin protein levels in t-DARPP expressing cells. In line with these findings, the knockdown of exogenous and endogenous t-DARPP led to a dramatic reduction of p-GSK-3β (Ser9) and β-catenin protein levels. These results support our hypothesis that t-DARPP regulates TCF/β-catenin activity through GSK-3β phosphorylation.

The phosphatidylinositol 3-kinase (PI3K)/AKT signaling pathway is a major regulator of GSK-3β where AKT phosphorylates and inactivates GSK-3β [39,48,66,67]. In this study, we demonstrated the regulation of phospho-AKT levels by t-DARPP, and confirmed that by using a PI3K/AKT pharmacologic inhibitor (LY294002) that t-DARPP-mediated activation of β-catenin is AKT-dependent. Previous reports suggested that t-DARPP provides anti-apoptotic and chemotherapeutic resistance properties to cancer cells through the activation of AKT and up-regulation of Bcl2 [17,18,68]. Taken together, the regulation of AKT by t-DARPP appears to be critical for several oncogenic functions in cancer cells.

## Conclusions

Our findings underscore a novel oncogenic function for t-DARPP in cancer cells through regulating the β-catenin/TCF cell signaling. Further studies are necessary to explore the full impact of t-DARPP signaling mechanisms in the development and progression of gastrointestinal malignancies.

## Methods

### Cell lines

AGS, MKN28, MKN45, and FLO-1 cell lines were purchased from American Type Culture Collection (Manassas, VA, USA). Cells were cultured in F-12 (HAM) medium supplemented with 5% penicillin-streptomycin (GIBCO, Grand Island, NY, USA) and 10% fetal bovine serum (Invitrogen Life Technologies, Carlsbad, CA, USA) in a 37°C incubator with an atmosphere containing 5% CO_2_. The pcDNA3.1 mammalian expression vector (Invitrogen) was used to generate a t-DARPP expression vector, as reported earlier [17]. AGS cell lines stably expressing t-DARPP or pcDNA3 empty vector were generated by transfection with respective expression plasmids using Lipofectamine 2000 (Invitrogen) followed by selection with 400 μg/mL of G418 antibiotic (Mediatech, Cellgro, Manassas, VA, USA) for three weeks. Stably transfected MKN28 and FLO-1 cell lines expressing t-DARPP were generated as described above, following selection with 600 μg/mL of G418 antibiotic. Single resistant colonies expressing t-DARPP were screened by Western blot analysis. Tetracycline inducible AGS cell line for t-DARPP was generated as described previously [68]. rtTA expression plasmid (Tet-On) was stably transfected into the AGS cell line using 20 μg of *ScaI *digested rtTA plasmid DNA. Single colonies stably expressing rtTA were selected using 400 μg/mL of G418. Following isolation, such colonies were transfected with pTRE-t-DARPP plasmid and selected with 0.8 μg/mL puromycin. Tet-responsive AGS cells stably expressing t-DARPP after induction with 2 μg/mL doxycycline (Clontech, Mountain View, CA, USA) were selected and examined with Western blot analysis.

### Luciferase assays

TCF luciferase reporter gene constructs, pTopFlash and its mutant pFopFlash were purchased from Upstate Biotechnology (Waltham, MA, USA). Renilla luciferase (Rluc) was inserted into pcDNA3.1 vector (Invitrogen) and expressed under the control of the CMV promoter. AGS, MKN28, and FLO-1 cells (5 × 10^4^) were plated in 24-well plates and transiently transfected with 500 ng of different combinations of pTopFlash, pFopFlash, pcDNA3-t-DARPP, pcDNA3 (empty vector), and 5 ng of Rluc using Fugene-6 (Roche Applied Science, Indianapolis, IN, USA) following manufacturer's protocol. Cells were lysed 48 h post-transfection and the assays for firefly luciferase activity and Renilla luciferase activity were performed using a luminometer (Turner Designs model TD20/20). The firefly luciferase activity was normalized to Renilla luciferase activity and expressed as relative luciferase activity.

### Immunofluorescence assay

AGS and MKN28 cells stably expressing t-DARPP or control vector and FLO-1 cells transiently expressing t-DARPP or control vector, were seeded onto an 8-chamber culture slide (BD Falcon, Bedford, MA, USA) (3 × 10^4 ^cells per chamber). After 24 h, the culture media was removed and cells were fixed in fresh 4% paraformaldehyde solution for one hour. Cells were then washed twice with cold PBS for one minute and permeabilized on ice for two minutes. After two washes with PBS, cells were incubated with 10% non-immune goat serum blocking solution (Zymed Laboratories, Carlsbad, CA, USA) for 20 min in a humidified chamber at room temperature. Next, cells were incubated with the β-catenin primary antibody (Sigma-Aldrich, St. Louis, MO, USA) prepared in PBS (1:200 dilution) for 2 h at room temperature, followed by three washes with PBS. Cells were then incubated with secondary affinipure donkey anti-rabbit IgG (Jackson Immunoresearch, West Grove, PA, USA) conjugated with fluorescein isothiocyanate (FITC) green fluorescence label prepared in PBS (1:1000 dilution) for 45 min at room temperature in a dark humidified chamber. Following three washes with PBS, cells were mounted using Vectashield/DAPI (Vector Laboratories, Burlingame, CA, USA) and visualized under a fluorescence microscope (Olympus Co., Tokyo, Japan). For analysis, all images were viewed and randomly captured at 40× magnification. For quantification, ImageJ software was used. The images were transformed into 8-bit and a region of interest (ROI) was randomly selected in the nucleus and cytoplasm. The ratio of integrated density in nucleus versus cytoplasm was determined by measuring the density of the ROI in the nucleus and cytoplasm. The percentage of cells that show β-catenin nuclear staining was determined based on the value of the density ratio; a value equal to or less than 1 was considered negative, a value more than 1 was considered positive.

### Western blot analysis

Protein lysates were prepared by scraping cultured cells in ice cold 1× PBS followed by centrifugation at 3500 rpm at 4°C for 10 min. Resulting protein pellets were suspended in cell lysis buffer (1% Triton X-100) containing 1% Halt protease/phosphatase inhibitor cocktail (Pierce, Rockford, IL, USA). Protein concentration was measured by a Bradford assay (Bio-Rad Laboratories, Hercules, CA, USA). Proteins (10 μg/lane) were separated by SDS/polyacrylamide gel electrophoresis and then transferred onto Hybond-P polyvinylidene diflouride membrane (Millipore, Bedford, MA, USA). Next, membranes were incubated with 5% non-fat dry milk blocking solution (Bio-Rad Laboratories) and target proteins were analyzed by incubating with primary antibodies specific to the proteins tested (Cell Signaling, Inc., Beverly, MA, USA).

### EDU cell proliferation assay

Cell proliferation was measured using the ClickiT^® ^EdU (5-ethynyl-2'-deoxyuridine) Assay (Invitrogen) which is a specific assay that measures actively proliferating cells. EdU is incorporated as thymidine analog in the DNA of newly dividing cells and is detected by a copper catalyzed reaction with Alexa Fluor 488 dye (green fluorescence). AGS cells stably expressing t-DARPP or control vector pcDNA3 (1.5 × 10^4^) were cultured in 8-well culture slides for 48 h. EdU labeling was done by incubating cells with 10 μM EdU solution prepared in pre-warmed complete medium at 37°C in an atmosphere containing 5% CO_2 _for one hour. Cells were then fixed in 3.7% paraformaldehyde solution prepared in 1 × PBS for 15 min at room temperature followed by two washes with 3% BSA in PBS. Next, cells were permeabilized by treating with permeabilization buffer (0.5% Triton X-100 in PBS) for 20 min. After rinsing the cells with wash solution, cells were incubated with 1 × ClickiT^® ^reaction cocktail containing ClickiT^® ^reaction buffer, CuSO_4 _solution, 1 × ClickiT^® ^reaction buffer additive and Alexa Fluor 488 dye for 30 min at room temperature in a dark humidified chamber. Before visualizing under a fluorescence microscope (Olympus Co.) at 40× magnification; cells were washed twice with 3% BSA in PBS, and then mounted using Vectashield/DAPI (Vector Laboratories). All experiments were performed in triplicate and 500 cells were counted from each experiment. The percentage of cells with nuclear EdU staining was calculated and graphed.

### Knockdown by small-interfering RNA

Small-interfering oligonucleotides (siRNA) specific to targeting t-DARPP were designed using the unique sequence, 5UTR and exon 1, of t-DARPP. The t-DARPP siRNA and scrambled siRNA were designed and purchased from Integrated DNA Technology (Coralville, IA, USA). MKN45 cells (2 × 10^5^) were cultured in a 6-well plate and transfected with different siRNA's (described above), following the manufacturer's protocol (Santa Cruz Biotechnology, CA, USA).

### Pharmacologic inhibition of PI3K/AKT signaling

In order to confirm t-DARPP-mediated regulation of TCF/β-catenin activity via the PI3K/AKT pathway, we used LY294002 (2-(4-morpholinyl)-8-phenyl-4H-1-benzopyran-4-one) to specifically inhibit phosphatidylinositol 3-Kinase activity [69]. AGS cells stably expressing t-DARPP were treated with LY492002 (40 μM) for 30 min and 2 h, as shown in Figure 5.

### Statistical analysis

A two tailed student's t-test was used to compare the statistical difference between two groups and a one-way ANOVA Newman-Keuls Multiple Comparison Test was used to compare the differences between three groups or more. The results were expressed as the mean with SD. The differences were considered statistically significant when the p value was ≤ 0.05.

## Competing interests

The authors declare that they have no competing interests.

## Authors' contributions

BV was involved in planning and performing experiments related to reporter assays, Western blot and functional assays. She summarized the data, generated the figures and contributed in writing parts of the manuscript. SZ and MS performed cell cultures and generated some of the reagents that were used. They participated in summarizing the data. AB assisted in the design of the experiments and participated in writing the Discussion section of the manuscript. WER is the principal investigator and was also involved in the design of the study, interpretation of data, troubleshooting experiments, and supervising the work relevant to this report. He participated in the writing and organization of the manuscript. All authors read and approved the final manuscript.

## References

[B1] Jemal A Siegel R Xu J Ward E Cancer statistics, 2010 CA Cancer J Clin 2010 60 277 300 10.3322/caac.20073 20610543

[B2] Parkin DM Bray F Ferlay J Pisani P Global cancer statistics, 2002 CA Cancer J Clin 2005 55 74 108 10.3322/canjclin.55.2.74 15761078

[B3] Blot WJ Devesa SS Kneller RW Fraumeni JF Jr Rising incidence of adenocarcinoma of the esophagus and gastric cardia JAMA 1991 265 1287 1289 10.1001/jama.265.10.1287 1995976

[B4] Pera M Epidemiology of esophageal cancer, especially adenocarcinoma of the esophagus and esophagogastric junction Recent Results Cancer Res 2000 155 1 14 10693234 10.1007/978-3-642-59600-1_1

[B5] Stein HJ Feith M Siewert JR Cancer of the esophagogastric junction Surg Oncol 2000 9 35 41 10.1016/S0960-7404(00)00021-9 11525305

[B6] Spechler SJ Barrett's esophagus and esophageal adenocarcinoma: pathogenesis, diagnosis, and therapy Med Clin North Am 2002 86 1423 1445 vii 10.1016/S0025-7125(02)00082-2 12510459

[B7] Lee W Patel JH Lockhart AC Novel targets in esophageal and gastric cancer: beyond antiangiogenesis Expert Opin Investig Drugs 2009 18 1351 1364 10.1517/13543780903179286 19642951

[B8] Polk DB Peek RM Jr Helicobacter pylori: gastric cancer and beyond Nat Rev Cancer 2010 10 403 414 10.1038/nrc2857 20495574 PMC2957472

[B9] Tahara E Genetic pathways of two types of gastric cancer IARC Sci Publ 2004 327 349 15055305

[B10] Zhang D Fan D New insights into the mechanisms of gastric cancer multidrug resistance and future perspectives Future Oncol 2010 6 527 537 10.2217/fon.10.21 20373867

[B11] Zhang D Fan D Multidrug resistance in gastric cancer: recent research advances and ongoing therapeutic challenges Expert Rev Anticancer Ther 2007 7 1369 1378 10.1586/14737140.7.10.1369 17944563

[B12] Greengard P The neurobiology of slow synaptic transmission Science 2001 294 1024 1030 10.1126/science.294.5544.1024 11691979

[B13] Varis A Zaika A Puolakkainen P Nagy B Madrigal I Kokkola A Vayrynen A Karkkainen P Moskaluk C El-Rifai W Knuutila S Coamplified and overexpressed genes at ERBB2 locus in gastric cancer Int J Cancer 2004 109 548 553 10.1002/ijc.20001 14991576

[B14] Beckler A Moskaluk CA Zaika A Hampton GM Powell SM Frierson HF Jr El-Rifai W Overexpression of the 32-kilodalton dopamine and cyclic adenosine 3',5'-monophosphate-regulated phosphoprotein in common adenocarcinomas Cancer 2003 98 1547 1551 10.1002/cncr.11654 14508844

[B15] Wang J Pan YL Liu N Guo CC Hong L Fan DM [Expression and significance of DARPP-32 in gastric carcinoma] Zhonghua Bing Li Xue Za Zhi 2004 33 350 353 15363322

[B16] Kauraniemi P Kuukasjarvi T Sauter G Kallioniemi A Amplification of a 280-kilobase core region at the ERBB2 locus leads to activation of two hypothetical proteins in breast cancer Am J Pathol 2003 163 1979 1984 10.1016/S0002-9440(10)63556-0 14578197 PMC1892409

[B17] Belkhiri A Zaika A Pidkovka N Knuutila S Moskaluk C El-Rifai W Darpp-32: a novel antiapoptotic gene in upper gastrointestinal carcinomas Cancer Res 2005 65 6583 6592 10.1158/0008-5472.CAN-05-1433 16061638

[B18] Belkhiri A Dar AA Peng DF Razvi MH Rinehart C Arteaga CL El-Rifai W Expression of t-DARPP mediates trastuzumab resistance in breast cancer cells Clin Cancer Res 2008 14 4564 4571 10.1158/1078-0432.CCR-08-0121 18579663 PMC2842884

[B19] Caspi M Zilberberg A Eldar-Finkelman H Rosin-Arbesfeld R Nuclear GSK-3beta inhibits the canonical Wnt signalling pathway in a beta-catenin phosphorylation-independent manner Oncogene 2008 27 3546 3555 10.1038/sj.onc.1211026 18223684

[B20] Giles RH van Es JH Clevers H Caught up in a Wnt storm: Wnt signaling in cancer Biochim Biophys Acta 2003 1653 1 24 12781368 10.1016/s0304-419x(03)00005-2

[B21] Kikuchi A Kishida S Yamamoto H Regulation of Wnt signaling by protein-protein interaction and post-translational modifications Exp Mol Med 2006 38 1 10 16520547 10.1038/emm.2006.1

[B22] Peifer M Sweeton D Casey M Wieschaus E wingless signal and Zeste-white 3 kinase trigger opposing changes in the intracellular distribution of Armadillo Development 1994 120 369 380 8149915 10.1242/dev.120.2.369

[B23] Papkoff J Rubinfeld B Schryver B Polakis P Wnt-1 regulates free pools of catenins and stabilizes APC-catenin complexes Mol Cell Biol 1996 16 2128 2134 8628279 10.1128/mcb.16.5.2128 PMC231200

[B24] Wodarz A Nusse R Mechanisms of Wnt signaling in development Annu Rev Cell Dev Biol 1998 14 59 88 10.1146/annurev.cellbio.14.1.59 9891778

[B25] Morin PJ Sparks AB Korinek V Barker N Clevers H Vogelstein B Kinzler KW Activation of beta-catenin-Tcf signaling in colon cancer by mutations in beta-catenin or APC Science 1997 275 1787 1790 10.1126/science.275.5307.1787 9065402

[B26] Damalas A Ben-Ze'ev A Simcha I Shtutman M Leal JF Zhurinsky J Geiger B Oren M Excess beta-catenin promotes accumulation of transcriptionally active p53 EMBO J 1999 18 3054 3063 10.1093/emboj/18.11.3054 10357817 PMC1171387

[B27] Polakis P The oncogenic activation of beta-catenin Curr Opin Genet Dev 1999 9 15 21 10.1016/S0959-437X(99)80003-3 10072352

[B28] Korinek V Barker N Morin PJ van Wichen D de Weger R Kinzler KW Vogelstein B Clevers H Constitutive transcriptional activation by a beta-catenin-Tcf complex in APC-/- colon carcinoma Science 1997 275 1784 1787 10.1126/science.275.5307.1784 9065401

[B29] Bian YS Osterheld MC Bosman FT Fontolliet C Benhattar J Nuclear accumulation of beta-catenin is a common and early event during neoplastic progression of Barrett esophagus Am J Clin Pathol 2000 114 583 590 10.1309/3QLC-5MF1-JYXU-A5XX 11026105

[B30] Rubinfeld B Albert I Porfiri E Fiol C Munemitsu S Polakis P Binding of GSK3beta to the APC-beta-catenin complex and regulation of complex assembly Science 1996 272 1023 1026 10.1126/science.272.5264.1023 8638126

[B31] Ikeda S Kishida S Yamamoto H Murai H Koyama S Kikuchi A Axin, a negative regulator of the Wnt signaling pathway, forms a complex with GSK-3beta and beta-catenin and promotes GSK-3beta-dependent phosphorylation of beta-catenin EMBO J 1998 17 1371 1384 10.1093/emboj/17.5.1371 9482734 PMC1170485

[B32] Sakanaka C Weiss JB Williams LT Bridging of beta-catenin and glycogen synthase kinase-3beta by axin and inhibition of beta-catenin-mediated transcription Proc Natl Acad Sci USA 1998 95 3020 3023 10.1073/pnas.95.6.3020 9501208 PMC19687

[B33] Komiya Y Habas R Wnt signal transduction pathways Organogenesis 2008 4 68 75 10.4161/org.4.2.5851 19279717 PMC2634250

[B34] Gordon MD Nusse R Wnt signaling: multiple pathways, multiple receptors, and multiple transcription factors J Biol Chem 2006 281 22429 22433 10.1074/jbc.R600015200 16793760

[B35] He X Semenov M Tamai K Zeng X LDL receptor-related proteins 5 and 6 in Wnt/beta-catenin signaling: arrows point the way Development 2004 131 1663 1677 10.1242/dev.01117 15084453

[B36] Munemitsu S Albert I Rubinfeld B Polakis P Deletion of an amino-terminal sequence beta-catenin in vivo and promotes hyperphosporylation of the adenomatous polyposis coli tumor suppressor protein Mol Cell Biol 1996 16 4088 4094 8754807 10.1128/mcb.16.8.4088 PMC231405

[B37] Yost C Torres M Miller JR Huang E Kimelman D Moon RT The axis-inducing activity, stability, and subcellular distribution of beta-catenin is regulated in Xenopus embryos by glycogen synthase kinase 3 Genes Dev 1996 10 1443 1454 10.1101/gad.10.12.1443 8666229

[B38] Aberle H Bauer A Stappert J Kispert A Kemler R beta-catenin is a target for the ubiquitin-proteasome pathway EMBO J 1997 16 3797 3804 10.1093/emboj/16.13.3797 9233789 PMC1170003

[B39] Pap M Cooper GM Role of glycogen synthase kinase-3 in the phosphatidylinositol 3-Kinase/Akt cell survival pathway J Biol Chem 1998 273 19929 19932 10.1074/jbc.273.32.19929 9685326

[B40] Jope RS Johnson GV The glamour and gloom of glycogen synthase kinase-3 Trends Biochem Sci 2004 29 95 102 10.1016/j.tibs.2003.12.004 15102436

[B41] Behrens J von Kries JP Kuhl M Bruhn L Wedlich D Grosschedl R Birchmeier W Functional interaction of beta-catenin with the transcription factor LEF-1 Nature 1996 382 638 642 10.1038/382638a0 8757136

[B42] Huber O Korn R McLaughlin J Ohsugi M Herrmann BG Kemler R Nuclear localization of beta-catenin by interaction with transcription factor LEF-1 Mech Dev 1996 59 3 10 10.1016/0925-4773(96)00597-7 8892228

[B43] Brunner E Peter O Schweizer L Basler K pangolin encodes a Lef-1 homologue that acts downstream of Armadillo to transduce the Wingless signal in Drosophila Nature 1997 385 829 833 10.1038/385829a0 9039917

[B44] He TC Sparks AB Rago C Hermeking H Zawel L da Costa LT Morin PJ Vogelstein B Kinzler KW Identification of c-MYC as a target of the APC pathway Science 1998 281 1509 1512 10.1126/science.281.5382.1509 9727977

[B45] Shtutman M Zhurinsky J Simcha I Albanese C D'Amico M Pestell R Ben-Ze'ev A The cyclin D1 gene is a target of the beta-catenin/LEF-1 pathway Proc Natl Acad Sci USA 1999 96 5522 5527 10.1073/pnas.96.10.5522 10318916 PMC21892

[B46] Tetsu O McCormick F Beta-catenin regulates expression of cyclin D1 in colon carcinoma cells Nature 1999 398 422 426 10.1038/18884 10201372

[B47] Yamada T Takaoka AS Naishiro Y Hayashi R Maruyama K Maesawa C Ochiai A Hirohashi S Transactivation of the multidrug resistance 1 gene by T-cell factor 4/beta-catenin complex in early colorectal carcinogenesis Cancer Res 2000 60 4761 4766 10987283

[B48] Beurel E Jope RS The paradoxical pro- and anti-apoptotic actions of GSK3 in the intrinsic and extrinsic apoptosis signaling pathways Prog Neurobiol 2006 79 173 189 10.1016/j.pneurobio.2006.07.006 16935409 PMC1618798

[B49] Baryawno N Sveinbjornsson B Eksborg S Chen CS Kogner P Johnsen JI Small-molecule inhibitors of phosphatidylinositol 3-kinase/Akt signaling inhibit Wnt/beta-catenin pathway cross-talk and suppress medulloblastoma growth Cancer Res 2010 70 266 276 10.1158/0008-5472.CAN-09-0578 20028853

[B50] Chalhoub N Baker SJ PTEN and the PI3-kinase pathway in cancer Annu Rev Pathol 2009 4 127 150 10.1146/annurev.pathol.4.110807.092311 18767981 PMC2710138

[B51] Hennessy BT Smith DL Ram PT Lu Y Mills GB Exploiting the PI3K/AKT pathway for cancer drug discovery Nat Rev Drug Discov 2005 4 988 1004 10.1038/nrd1902 16341064

[B52] Luo HR Hattori H Hossain MA Hester L Huang Y Lee-Kwon W Donowitz M Nagata E Snyder SH Akt as a mediator of cell death Proc Natl Acad Sci USA 2003 100 11712 11717 10.1073/pnas.1634990100 14504398 PMC208823

[B53] El-Rifai W Smith MF Jr Li G Beckler A Carl VS Montgomery E Knuutila S Moskaluk CA Frierson HF Jr Powell SM Gastric cancers overexpress DARPP-32 and a novel isoform, t-DARPP Cancer Res 2002 62 4061 4064 12124342

[B54] Varis A Wolf M Monni O Vakkari ML Kokkola A Moskaluk C Frierson H Jr Powell SM Knuutila S Kallioniemi A El-Rifai W Targets of gene amplification and overexpression at 17q in gastric cancer Cancer Res 2002 62 2625 2629 11980659

[B55] Maqani N Belkhiri A Moskaluk C Knuutila S Dar AA El-Rifai W Molecular dissection of 17q12 amplicon in upper gastrointestinal adenocarcinomas Mol Cancer Res 2006 4 449 455 10.1158/1541-7786.MCR-06-0058 16849520

[B56] Hemmings HC Jr Nairn AC Aswad DW Greengard P DARPP-32, a dopamine- and adenosine 3':5'-monophosphate-regulated phosphoprotein enriched in dopamine-innervated brain regions. II. Purification and characterization of the phosphoprotein from bovine caudate nucleus J Neurosci 1984 4 99 110 6319628 10.1523/JNEUROSCI.04-01-00099.1984 PMC6564759

[B57] Hemmings HC Jr Nairn AC McGuinness TL Huganir RL Greengard P Role of protein phosphorylation in neuronal signal transduction FASEB J 1989 3 1583 1592 2493406 10.1096/fasebj.3.5.2493406

[B58] Vangamudi B Peng DF Cai Q El-Rifai W Zheng W Belkhiri A t-DARPP regulates phosphatidylinositol-3-kinase-dependent cell growth in breast cancer Mol Cancer 9 240 10.1186/1476-4598-9-240 20836878 PMC2945963

[B59] Polakis P Wnt signaling and cancer Genes Dev 2000 14 1837 1851 10921899

[B60] Logan CY Nusse R The Wnt signaling pathway in development and disease Annu Rev Cell Dev Biol 2004 20 781 810 10.1146/annurev.cellbio.20.010403.113126 15473860

[B61] Washington K Chiappori A Hamilton K Shyr Y Blanke C Johnson D Sawyers J Beauchamp D Expression of beta-catenin, alpha-catenin, and E-cadherin in Barrett's esophagus and esophageal adenocarcinomas [In Process Citation] Mod Pathol 1998 11 805 813 9758359

[B62] Clements WM Wang J Sarnaik A Kim OJ MacDonald J Fenoglio-Preiser C Groden J Lowy AM beta-Catenin mutation is a frequent cause of Wnt pathway activation in gastric cancer Cancer Res 2002 62 3503 3506 12067995

[B63] Crawford HC Fingleton BM Rudolph-Owen LA Goss KJ Rubinfeld B Polakis P Matrisian LM The metalloproteinase matrilysin is a target of beta-catenin transactivation in intestinal tumors Oncogene 1999 18 2883 2891 10.1038/sj.onc.1202627 10362259

[B64] Moon RT Bowerman B Boutros M Perrimon N The promise and perils of Wnt signaling through beta-catenin Science 2002 296 1644 1646 10.1126/science.1071549 12040179

[B65] Takahashi-Yanaga F Sasaguri T GSK-3beta regulates cyclin D1 expression: a new target for chemotherapy Cell Signal 2008 20 581 589 10.1016/j.cellsig.2007.10.018 18023328

[B66] Mitsiades CS Mitsiades N Koutsilieris M The Akt pathway: molecular targets for anti-cancer drug development Curr Cancer Drug Targets 2004 4 235 256 10.2174/1568009043333032 15134532

[B67] Sourbier C Lindner V Lang H Agouni A Schordan E Danilin S Rothhut S Jacqmin D Helwig JJ Massfelder T The phosphoinositide 3-kinase/Akt pathway: a new target in human renal cell carcinoma therapy Cancer Res 2006 66 5130 5142 10.1158/0008-5472.CAN-05-1469 16707436

[B68] Belkhiri A Dar AA Zaika A Kelley M El-Rifai W t-Darpp promotes cancer cell survival by up-regulation of Bcl2 through Akt-dependent mechanism Cancer Res 2008 68 395 403 10.1158/0008-5472.CAN-07-1580 18199533

[B69] Vlahos CJ Matter WF Hui KY Brown RF A specific inhibitor of phosphatidylinositol 3-kinase, 2-(4-morpholinyl)-8-phenyl-4H-1-benzopyran-4-one (LY294002) J Biol Chem 1994 269 5241 5248 8106507

